# Effects of triple therapy on disease burden in patients of GOLD groups C and D: results from the observational COPD cohort COSYCONET

**DOI:** 10.1186/s12890-024-02902-4

**Published:** 2024-03-01

**Authors:** Jennifer A. Zader, Rudolf A. Jörres, Imke Mayer, Peter Alter, Robert Bals, Henrik Watz, Pontus Mertsch, Klaus F. Rabe, Felix Herth, Franziska C. Trudzinski, Tobias Welte, Hans-Ulrich Kauczor, Jürgen Behr, Julia Walter, Claus F. Vogelmeier, Kathrin Kahnert

**Affiliations:** 1 Berlin School of Public Health, Charité Universitätsmedizin Berlin, Berlin, Germany; 2 Institute and Outpatient Clinic for Occupational, Social and Environmental Medicine, Comprehensive Pneumology Center Munich (CPC-M), Ludwig-Maximilians-Universität München, Munich, Germany; 3 Institute of Public Health, Charité Universitätsmedizin Berlin, Berlin, Germany; 4 PreMeDICaL, Inria Montpellier, IDESP, Montpellier, France; 5 Department of Medicine, Pulmonary and Critical Care Medicine, German Center for Lung Research (DZL), University Medical Center Giessen and Marburg, Philipps-University Marburg, Germany, Marburg, Germany; 6 Department of Internal Medicine V – Pulmonology, Allergology, Respiratory Intensive Care Medicine, Saarland University Hospital, Kirrberger Straße 1, 66424 Homburg, Germany; 7 Helmholtz Centre for Infection Research (HZI), Helmholtz Institute for Pharmaceutical Research Saarland (HIPS), Saarland University Campus, 66123 Saarbrücken, Germany; 8 Member of the German Center for Lung Research, Pulmonary Research Institute at Lung Clinic Grosshansdorf, Airway Research Center North, Woehrendamm 80, 22927 Grosshansdorf, Germany; 9 Department of Medicine V, Comprehensive Pneumology Center, Member of the German Center for Lung Research (DZL), University Hospital, LMU Munich, Ziemssenstr.1, 80336 Munich, Germany; 10 Faculty of Medicine, Christian-Albrechts-Universität Zu Kiel, 24098 Kiel, Germany; 11 Thoraxklinik-Heidelberg gGmbH, Röntgenstraße 1, 69126 Heidelberg, Germany; 12 Member of the German Center for Lung Research, Translational Lung Research Centre Heidelberg (TLRC), Heidelberg, Germany; 13 Department of Pneumology, Hannover Medical School, Carl-Neuberg-Str. 1, 30625 Hannover, Germany; 14 Department of Diagnostic & Interventional Radiology, University Hospital of Heidelberg, Heidelberg, Germany; 15 Department of Radiology, German Cancer Research Center (DKFZ), Heidelberg, Germany

**Keywords:** COPD, Triple therapy, Symptoms, Lung function, Health care costs

## Abstract

**Background:**

Randomized controlled trials described beneficial effects of inhaled triple therapy (LABA/LAMA/ICS) in patients with chronic obstructive pulmonary disease (COPD) and high risk of exacerbations. We studied whether such effects were also detectable under continuous treatment in a retrospective observational setting.

**Methods:**

Data from baseline and 18-month follow-up of the COPD cohort COSYCONET were used, including patients categorized as GOLD groups C/D at both visits (*n* = 258). Therapy groups were defined as triple therapy at both visits (triple always, TA) versus its complement (triple not always, TNA). Comparisons were performed via multiple regression analysis, propensity score matching and inverse probability weighting to adjust for differences between groups. For this purpose, variables were divided into predictors of therapy and outcomes.

**Results:**

In total, 258 patients were eligible (TA: *n* = 162, TNA: *n* = 96). Without adjustments, TA patients showed significant (*p* < 0.05) impairments regarding lung function, quality of life and symptom burden. After adjustments, most differences in outcomes were no more significant. Total direct health care costs were reduced but still elevated, with inpatient costs much reduced, while costs of total and respiratory medication only slightly changed.

**Conclusion:**

Without statistical adjustment, patients with triple therapy showed multiple impairments as well as elevated treatment costs. After adjusting for differences between treatment groups, differences were reduced. These findings are compatible with beneficial effects of triple therapy under continuous, long-term treatment, but also demonstrate the limitations encountered in the comparison of controlled intervention studies with observational studies in patients with severe COPD using different types of devices and compounds.

**Supplementary Information:**

The online version contains supplementary material available at 10.1186/s12890-024-02902-4.

## Background

Chronic obstructive pulmonary disease (COPD) is a common respiratory disease worldwide and associated with high morbidity and mortality [1]. Its treatment by inhaled medication is primarily related to the magnitude and frequency of symptoms and exacerbations, according to recommendations of the international GOLD consortium [1] and its national version [2]. They all describe a strategy of therapeutic escalation, starting from long-acting beta-agonists (LABA) and long-acting muscarinic antagonists (LAMA), with a potential step-up via inhaled corticosteroids (ICS). The proposed combinations include LABA + LAMA, LABA + ICS and LABA + LAMA + ICS (triple therapy). According to GOLD 2022 [1] and other sources [2], ICS and triple therapy are recommended only for patients with a high risk of exacerbations and hospitalization (GOLD groups C/D). This is based on the results of randomized controlled trials (RCT) that demonstrated positive effects of triple therapy on the course of the disease including the rate of exacerbations and mortality [3–8]. Favorable effects of triple therapy on mortality were also shown in comparison to other combination therapies such as LABA + LAMA or LABA + ICS, at least regarding the first three months of use [5, 6], although recent data indicate a decline of this effect during longer follow-up [9, 10].

RCTs are known for their limitation of being selective regarding the enrolled patients, due to the application of well-defined inclusion and exclusion criteria. As shown for COPD [11, 12], they typically comprise a high proportion of patients with low comorbidity burden and/or above-average performance indices and thus do not necessarily represent the spectrum of patients encountered in daily clinical practice. Consequently, both the prescription and the observable effects of medication could be different in real-life situations, a factor that might contribute to the reported discrepancies between actual COPD medication and recommendations [13]. Observational studies probably suffer less from inclusion bias, however, being not randomized they are prone to other types of bias, such as the fact that the likelihood of medication depends on the severity of disease. Still, as they are certainly closer to real-life conditions, it is of interest which effects of medication can be identified. In a previous study on the (not recommended) use of ICS in GOLD A/B patients, we found that patients with ICS had more severe disease than those without, as indicated by a variety of clinical and functional parameters [14]. After statistical post hoc-matching, the differences between treatment groups vanished or were partially reversed. In analogy, we expected that in patients of GOLD groups C/D triple therapy would be associated with more severe disease. Using post-hoc matching it should be possible to reveal potential beneficial effects of triple therapy, or at least whether the differences would be reduced or vanish, indicating a tendency for beneficial effects. The result would also be of methodological interest regarding the potential and limits of purely observational data in COPD. It was the aim of the present study to perform such an analysis, using data from the prospective multi-center observational COPD cohort COSYCONET (COPD and SYstemic consequences-COmorbidities NETwork) [15]. COSYCONET aims at investigating the interaction between lung disease, comorbidities and systemic inflammation under real-world conditions; the recruitment took place between 2010 and 2013 in 31 study centers all over Germany [15].

## Methods

### Study population

Data from visit 1 (baseline, *n* = 2741) and visit 3 (18-month follow-up, *n* = 2053) of the COSYCONET cohort were used. These visits took place in 2010 until 2015. Further information about the study and its inclusion/exclusion criteria can be found elsewhere [15]. Patients were categorized into GOLD grades according to spirometry and into GOLD groups A-D according to exacerbations and symptoms [1], using the modified Medical Research Council (mMRC) questionnaire. The present analysis was restricted to patients of grades 1–4 at visit 1 (*n* = 2291) and further limited to those categorized as groups C or D at visits 1 and 3 (*n* = 268) in order to satisfy the formal criteria for triple therapy at both visits. The requirement of completeness of data resulted in the exclusion of 10 patients and thus a final study population of 258 patients, among them 96 of group C and 162 of group D. The protocol of COSYCONET was approved by the ethical committees of all study centers, and all patients gave their written informed consent. The study is registered under the identifier NCT01245933 (first registration 23/11/2010). COSYCONET was approved by the ethics committees of all study centers and conducted in accordance with the Declaration of Helsinki.

### Assessments

Lung function measurements included spirometry, body plethysmography and diffusing capacity for carbon monoxide (CO), yielding values for forced expiratory volume in 1 s (FEV_1_), forced vital capacity (FVC), their ratio (FEV_1_/FVC), intrathoracic gas volume (ITGV), the ratio (RV/TLC) of residual volume (RV) to total lung capacity (TLC), transfer factor (TLCO) and transfer coefficient (KCO) for CO. Predicted values were taken from established sources [16–18]. Blood gas analysis provided the partial pressures of oxygen (PaO_2_) and carbon dioxide (PaCO_2_) as well as oxygen saturation (SaO_2_), determined from the hyperemic ear lobe.

Assessments further comprised the determination of age, height, body-mass index (BMI), years since COPD diagnosis and 6-min walking distance (6-MWD). Furthermore, we used the International Physical Activity Questionnaire (IPAQ) [19], the St. George’s Respiratory Questionnaire (SGRQ) with its sub-scores activity, symptoms and impact, the PHQ-9 questionnaire on depression, an analog scale on generic quality of life (EQ-5D VAS) [20], and the COPD Assessment Test (CAT) the results of which were analyzed per single item. Educational status was categorized into three groups based on the number of completed years of education (basic ≤ 9 years, secondary 10 to 11 years, higher > 11 years). Data on comorbidities were obtained via patients’ reports of physician-based diagnoses [21]. Details of all assessments have been given previously [15]. The spectrum of questionnaires as well as lung function and physical capacity was chosen in order to characterize the clinical burden of the disease in a comprehensive manner, in addition to the economic burden derived from a cost calculation.

### Cost calculation

The analysis of annual health care costs data included prescription-only pharmaceuticals, based on name, daily defined doses (ddd), pharmacy retail prices and national drug code. Costs were calculated by multiplying in- and outpatient, physiotherapy and rehabilitation items with German unit costs and winsorized at the 95th percentile [14]. Data refer to the price year of 2012, i.e. the time when the assessments were performed.

### Medication

All patients were asked to bring their medication to each study visit. Respiratory medication was categorized according to its compounds and combinations into LABA, LAMA, ICS, LABA + ICS, LAMA + ICS, LABA + LAMA, LABA + LAMA + ICS (triple), independently of the use of single or combined inhalers. For each single compound or combination, we defined its presence at visits 1 and 3 as “always”, the complementary category being “not always” (control). The term “not always” versus “always” does not refer to the daily intake of the medication and thus to adherence, but to the presence of the respective medication at the various study time points examined; in COSYCONET very high treatment adherence has been demonstrated [22]. The Supplemental Table S1 shows the distribution over these categories for the different types of medication. The “not always” group was largest for triple therapy and, in line with this, for LAMA + ICS. The alternative categorization into “always” versus “never” (neither at visit 1 nor at visit 3) was not considered, as the “never” group comprised only 43 patients. Concordant with the aim of our study, sample sizes supported our focus on triple therapy and the comparison of “always” with “not always”.

### Statistical analysis

Mean values and standard deviations (SD), median values and quartiles, or numbers and percentages were used for data description, depending on the type of data. Unadjusted comparisons between “always” versus “not always” triple therapy were performed using either the t-test/wilcoxon rank-sum test or contingency tables and chi-square/fisher exact statistics. To identify variables associated with the prescription of triple therapy, logistic regression analysis was used. The predictors tested comprised GOLD groups C versus D, sex, age, height, BMI, the diagnosis of asthma, smoking status (active versus non-active), education (basic, secondary, high), years of COPD diagnosis, FEV_1_%predicted, FEV_1_/FVC, RV/TLC, and TLCO %predicted. The predictors were chosen based on the assumption that in patients with high levels of exacerbations and/or symptoms functional parameters might have been particularly relevant for the therapeutic decisions by the treating physicians. For all functional variables, age, height and BMI, mean values of data from visit 1 and 3 were taken, whereas for sex, years of COPD diagnosis, GOLD C versus D, smoking status and education, data of visit 1 were taken.

To quantify the potential effect of triple therapy after matching, various outcome measures were used. These included FVC %predicted, ITGV %predicted, PaO_2_, PaCO_2_, SaO_2_, 6-MWD, IPAQ, St. George Respiratory Questionnaire impact, symptoms and activity scores, EQ-5D VAS, total CAT score and all single CAT items, as well as PHQ-9, all taken as mean values from visits 1 and 3. Cost variables comprised total direct costs, in- and outpatient costs, medication costs based on prescription of respiratory medication and other medication, and costs from rehabilitation and physiotherapy utilization.

Each of these outcome measures was analyzed in four different ways. First, an unadjusted comparison between the two treatment groups triple “always” versus triple “not always” was performed. Second, adjustment was done by linear regression analysis, always taking as predictors those given in Table 1. This resulted in adjusted effect estimates of triple therapy. As a third method, the same predictors and outcome variables were used in propensity score analyses, employing the optimal full matching procedure, which is well suited to deal with different, unbalanced groups [23, 24]. To quantify differences in mean outcomes between the treatment groups, we used the average treatment effect (ATE). Standardized mean differences were computed to evaluate the quality of matching, and differences < 0.1 were considered as indicative of successful matching [25, 26]. As an alternative (confounding) adjustment procedure, we used regression analysis with inverse probability weighting (IPW) [14].

**Table 1:** Patient characteristics used as predictors in adjustments and matching procedures

	**Triple always**	**Triple not always**	**All**
Number	162	96	258
GOLD group C (mMRC)	50 (30.9%)	39 (40.6%)	89 (34.5%)
GOLD group D (mMRC)	112 (69.1%)	57 (59.4%)^***^	169 (65.5%)
Age (y)	64.6 (± 7.55)	63.7 (± 7.86)	64.2 (± 7.66)
Male	91 (56.2%)	54 (56.3%)	145 (56.2%)
Female	71 (43.8%)	42 (43.8%)	113 (43.8%)
Height (cm)	169 (± 8.66)	171 (± 9.19)	170 (± 8.87)
BMI (kg/m^2^)	25.9 (± 4.84)	26.9 (± 5.58)	26.3 (± 5.14)
Basic education	99 (61.1%)	45 (46.9%)	144 (55.8%)
Secondary education	45 (27.8%)	36 (37.5%)	81 (31.4%)
Higher education	18 (11.1%)	15 (15.6%)	33 (12.8%)
Smoking status (active)	26 (16.0%)	23 (24.0%)	49 (19.0%)
Time since COPD diagnosis (y)	9.79 (± 7.56)	9.01 (± 7.87)	9.50 (± 7.67)
Asthma (yes)	40 (24.7%)	22 (22.9%)	62 (24.0%)
FEV_1_ (%predicted)	41.8 (± 14.2)	49.4 (± 17.3)^***^	44.7 (± 15.8)
FEV_1_/FVC (%)	60.4 (± 12.0)	64.3 (± 14.5)^*^	61.8 (± 13.1)
RV/TLC (%)	153 (± 25.2)	140 (± 25.9)^***^	148 (± 26.1)
TLCO (%predicted)	45.3 (± 19.3)	54.5 (± 20.9)^***^	48.7 (± 20.4)

Mean values and standard deviations or numbers and percentages are given. The groups “Triple always” and “Triple not always” were compared with each other using chi-square statistics/Fisher’s exact test for categorical variables and unpaired t-test/Wilcoxon rank-sum test for continuous variables. Significance levels are indicated as ^*^p < 0.05, ^**^p < 0.01, ^***^p < 0.001

These three approaches were chosen in order to compare the consistency of the results and to reduce the likelihood that conclusions were critically dependent on a specific statistical approach. We expected that patients with “triple always” would show clinical and functional impairment compared to patients “triple not always”, in analogy to our previous results regarding ICS in GOLD groups A and B [14]. The question would then be, whether the differences in outcome variables would become smaller, vanish or even be reverted to beneficial effects when adjusting for the differences between groups. Statistical significance was assumed for *p*-values < 0.05. All analyses were performed using the programming environment R and R Studio (version 4.0.3), specifically the package “MatchIt”[27].

## Results

### Baseline characteristics

Patient characteristics regarding the set of variables used as potential predictors are shown in Table 1. There were statistically significant (*p* < 0.05 each) differences between the groups, TA versus TNA regarding GOLD groups C versus D, FEV_1_%predicted, RV/TLC and TLCO %predicted. We also compared comorbidities; there were no significant differences, with average prevalence values across groups for hypertension being 55.4%, coronary artery disease 16.3%, heart failure 6.6%, previous cardiac infarction 7.4%, diabetes 12.8%, hyperlipidemia 37.6%, osteoporosis 23.3%, sleep apnea 15.5%, and asthma 24.0%. Only asthma was taken into the set of predictors, as it might be related to ICS therapy.

Further characteristics are given in Table 2; these variables were used as functional and clinical outcome measures. SGRQ activity score, 6-MWD, FVC %predicted and ITGV %predicted significantly (*p* < 0.05 each) differed between patients with triple therapy “always” versus “not always”. Data regarding annual health care costs are shown in Table 3. Unadjusted total direct costs, medication costs, costs for respiratory medication, outpatient cost and cost of physiotherapy significantly (*p* < 0.05 each) differed between the two treatment groups.

**Table 2:** Patient characteristics used as outcome measures after application of adjustment or matching procedures using the variables given in Table 1

	**Triple always**	**Triple not always**	**All**
Number	162	96	258
FVC (%predicted)	69.1 (± 16.9)	75.9 (± 16.4)^**^	71.6 (± 17.0)
ITGV (%predicted)	166 (± 37.5)	156 (± 35.8)^*^	162 (± 37.1)
PaCO_2_ (mmHg)	39.3 (± 4.79)	38.5 (± 4.37)	39.0 (± 4.65)
PaO_2_ (mmHg)	65.2 (± 7.98)	66.5 (± 8.46)	65.7 (± 8.17)
SaO_2_ (%)	93.5 (± 2.44)	93.8 (± 2.68)	93.6 (± 2.53)
IPAQ (score)	4020 (± 3960)	4300 (± 4020)	4120 (± 3970)
EQ-VAS (score)	49.7 (± 15.5)	52.5 (± 15.7)	50.7 (± 15.6)
SGRQ activity (score)	74.7 (± 20.3)	65.4 (± 22.2)^***^	71.3 (± 21.5)
SGRQ impact (score)	41.3 (± 19.1)	39.3 (± 17.4)	40.5 (± 18.5)
SGRQ symptoms (score)	66.9 (± 16.4)	65.6 (± 14.0)	66.4 (± 15.6)
6-MWD (m)	358 (± 98.4)	399 (± 115)^**^	374 (± 107)
PHQ-9 (score)	7.42 (± 4.58)	8.14 (± 4.56)	7.69 (± 4.58)
CAT1 cough (score)	2.56 (± 0.956)	2.70 (± 0.922)	2.61 (± 0.944)
CAT2 phlegm (score)	2.76 (± 1.06)	2.71 (± 1.05)	2.74 (± 1.06)
CAT3 chest tightness (score)	2.31 (± 1.25)	2.32 (± 1.11)	2.31 (± 1.20)
CAT4 breathlessness (score)	4.18 (± 0.780)	4.10 (± 0.908)	4.15 (± 0.829)
CAT5 activity (score)	3.12 (± 1.20)	2.82 (± 1.23)	3.01 (± 1.22)
CAT6 confidence (score)	1.66 (± 1.25)	1.41 (± 1.28)	1.57 (± 1.27)
CAT7 sleep (score)	2.98 (± 1.35)	2.65 (± 1.31)	2.55 (± 1.33)
CAT8 energy (score)	2.98 (± 0.958)	3.07 (± 0.983)	3.01 (± 0.967)
CAT Sum (score)	22.1 (± 6.25)	21.8 (± 6.03)	22.0 (± 6.16)

Mean values and standard deviations are given. The groups “Triple always” and “Triple not always” were compared with each other using the t-test or wilcoxon rank-sum test, depending on the distribution of the data. Significance levels are indicated as ^*^p < 0.05, ^**^p < 0.01, ^***^p < 0.001

**Table 3:** Outcome measures in terms of health care costs used after adjustment and matching using the variables given in Table 1

Annual health care costs (€)	Triple always	Triple not always	All
Number	162	96	258
Total direct costs	13600 (± 8960)	10800 (± 8700)^**^	12500 (± 8950)
Medication costs	3630 (± 1850)	2650 (± 1780)^***^	3260 (± 1880)
Respiratory medication costs	2050 (± 426)	1460 (± 531)^***^	1830 (± 548)
Other medication costs	1580 (± 1760)	1190 (± 1600)	1440 (± 1710)
Inpatient costs	963 (± 606)	926 (± 541)	949 (± 582)
Outpatient costs	5560 (± 4390)	4440 (± 4200)^*^	5140 (± 4350)
Rehabilitation costs	637 (± 814)	524 (± 775)	595 (± 800)
Physiotherapy costs	133 (± 188)	84.7 (± 145)^*^	115 (± 175)

Mean values and standard deviations are given. Medication costs were restricted to prescription-only pharmaceuticals and based on information about name, national drug code, defined daily doses, and pharmacy retails prices. All costs refer to the price year 2012. The groups “Triple always” and “Triple not always” were compared with each other using wilcoxon rank-sum test. Significance levels are indicated as ^*^p < 0.05, ^**^p < 0.01, ^***^p < 0.001

### Predictors of triple therapy

If considered separately, the results regarding differences of single predictor variables between treatment groups as indicated in Table 1 were confirmed by logistic regression analysis, using TA as dependent variable. If all predictor variables were taken into account simultaneously, there were no statistically significant associations, probably due to their degree of collinearity. If using several selection procedures, consistently RV/TLC was identified as the most relevant predictor (*p* < 0.001) of triple therapy, whereby higher values were associated with higher likelihood of triple therapy. Although regression modelling did not yield a significant result, the pre-selected potential predictors were not considered redundant because the selection was based on clinical availability and plausibility. Therefore, they were still used for further adjustment techniques.

### Associations between triple therapy and clinical and functional outcome measures

Unadjusted differences between the treatment groups, corresponding to the difference between mean values shown in Table 2, are given in the first data column of Table 4. As indicated by the confidence intervals, differences that were statistically different from zero were in accordance with the comparison of mean values given in Table 2.

**Table 4:** Pre-defined clinical and functional outcome measures analyzed by different statistical approaches regarding the comparison “Triple always” versus “Triple not always”

Outcome measure		Univariate	Linear Regression	Optimal full matching	IPW
**SGRQ activity (score)**	Estimate	9.625	3.197	1.835	2.611
	95%CI	4.009; 14.685	-0.778; 7.172	-4.811; 8.481	-3.253; 8.476
	*p* value	**< 0.001**	0.116	0.589	0.384
**SGRQ impact (score)**	Estimate	1.946	-0.357	-1.811	-0.728
	95%CI	-2.753; 6.644	-4.960 4.245	-7.583; 3.961	-5.943; 4.488
	*p* value	0.416	0.879	0.539	0.785
**SGRQ symptoms (score)**	Estimate	1.291	4.233e-01	-1.933	0.106
	95%CI	-2.664; 5.246	-3.589; 4.454	-6.559; 2.692	-4.197; -4.409
	p value	0.521	0.833	0.414	0.962
**IPAQ (score)**	Estimate	-281.629	245.720	-44.08	161.7
	95%CI	-1318.311; 755.072	-819.705; 1311.145	-1249.088; 1160.928	-901.404; 1224.804
	*p* value	0.593	0.652	0.943	0.766
**6-MWD (m)**	Estimate	-40.633	-9.252	-4.144	-8.116
	95%CI	68.553; 12.715	-31.672; 13.166	-38.246; 29.958	-40.022; 23.791
	*p* value	**0.0045**	0.419	0.812	0.619
**EQ-VAS (score)**	Estimate	-2.787	0.075	1.252	0.590
	95%CI	-6.739; 1.163	-3.504; -3.655	-3.948; 6.452	-4.116; 5.295
	*p* value	0.165	0.967	0.637	0.806
**FVC /%predicted)**	Estimate	-6.811	2.04e-01	-0.262	-0.019
	95%CI	-11.048; -2.573	-0.621; 1.029	-11.202; 10.679	-4.616; 4.579
	*p* value	**0.002**	0.627	0.919	0.994
**ITGV (% predicted)**	Estimate	9.625	-3.840	-5.342	-5.342
	95%CI	0.269; 18.981	-8.352; 0.672	-16.136; 5.452	-16.136; 5.452
	*p* value	**0.044**	0.097	0.333	0.333
**PHQ9 (score)**	Estimate	-0.724	-1.123	-1.555	-1.252
	95%CI	-1.892; 0.444	-2.267; 0.020	-2.932; -0.179	-2.647; 0.142
	*p* value	0.223	0.055	**0.028**	0.0796
**CAT1 cough (score)**	Estimate	-0.133	-0.116	-0.126	-0.097
	95%CI	-0.372; 0.106	-0.360; 0.129	-0.418; 0.165	-0.383; 0.189
	*p* value	0.274	0.354	0.396	0.506
**CAT2 phlegm (score)**	Estimate	0.054	0.008	-0.111	0.017
	95%CI	-0.214; 0.322	-0.265; 0.282	-0.430; 0.208	-0.303; 0.337
	*p* value	0.691	0.951	0.495	0.917
**CAT3 chest tightness (score)**	Estimate	-0.009	-0.129	-0.224	-0.187
	95%CI	-0.313; 0.295	-0.429; 0.172	-0.580; 0.132	-0.522; 0.149
	*p* value	0.953	0.402	0.219	0.276
**CAT4 breathlessness (score)**	Estimate	0.083	-0.077	-0.092	-0.070
	95%CI	-0.127; 0.294	-0.262; 0.109	-0.335; -0.151	-0.298; 0.158
	*p* value	0.437	0.418	0.46	0.549
**CAT5 activity (score)**	Estimate	0.300	0.018	-0.062	0.003
	95%CI	-0.007; 0.608	-0.242; 0.278	-0.398; 0.273	-0.309; 0.316
	*p* value	0.055	0.891	0.716	0.984
**CAT6 confidence (score)**	Estimate	0.254	-0.022	-0.247	-0.104
	95%CI	-0.066; 0.574	-0.322; 0.277	-0.665; 0.172	-0.488; 0.281
	*p* value	0.119	0.884	0.249	0.598
**CAT7 sleep (score)**	Estimate	-0.157	-0.080	-0.261	-0.210
	95%CI	-0.495; 0.181	-0.526; 0.154	-0.627; 0.105	-0.552; 0.133
	*p* value	0.361	0.284	0.164	0.231
**CAT8 energy (score)**	Estimate	-0.092	-0.139	-0.269	-0.150
	95%CI	-0.338; 0.153	-0.373; 0.095	-0.545; 0.006	-0.424; 0.125
	*p* value	0.459	0.245	0.056	0.285
**CAT Sum (score)**	Estimate	0.300	-0.642	-1.393	0.797
	95%CI	-1.264;1.864	-2.094; 0.810	-3.276; 0.490	-2.586; 0.992
	*p* value	0.706	0.387	0.148	0.383
**PaCO**_**2**_** (mmHg)**	Estimate	0.755	-0.335	-0.109	-0.252
	95%CI	-0.422; 1.931	-1.406; 0.735	-1.383; 1.166	-1.456; 0.951
	*p* value	0.207	0.540	0.867	0.681
**PaO**_**2**_** (mmHg)**	Estimate	-1.331	-0.380	-0.537	0.359
	95%CI	-3.401; 0.739	-2.415; 1.655	-3.067; 1.994	-2.544; 1.827
	*p* value	0.207	0.715	0.678	0.748
**SaO**_**2**_** (%)**	Estimate	-0.341	-0.052	0.073	-0.046
	95%CI	-0.991; 0.308	-0.693; 0.588	-0.993; 0.846	-0.822; 0.729
	*p* value	0.302	0.873	0.876	0.907

*IPW* Inverse Probability Weighting. For abbreviations of variables see text. All variables shown were first tested in univariate analyses and then in the multivariable analyses shown in the other three columns, always using the set of predictors shown in Table 1

The second column shows the results of multiple linear regression analysis taking into account the predictors of Table 1. There were no more statistically significant differences and the magnitude of differences was mostly reduced or even reversed in sign, compared to the unadjusted differences.

**Fig. 1:**
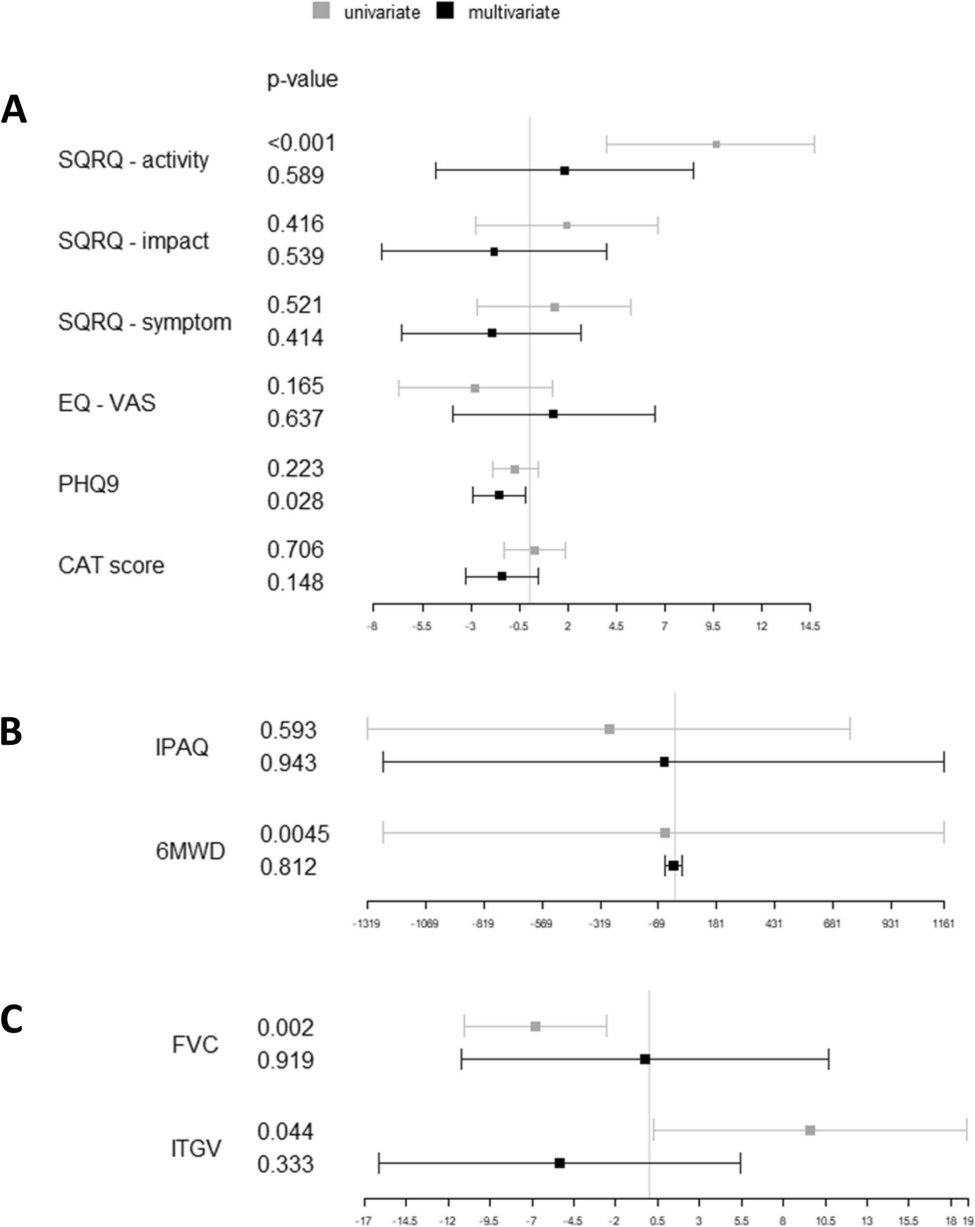
Results of univariate comparisons and optimal full matching based on the data given in Table 4. The grey rectangles show the effect estimates regarding TA versus TNA with their 95% confidence intervals. The black rectangles symbolize the estimate obtained after full matching for the predictors listed in Table 1. The results are presented in three different panels due to the need for different scaling. Panel A shows data from disease-specific quality of life (SGRQ), generic quality of life (EQ-VAS), a depression score (PHQ9), as well as the 8 items of COPD assessment test (CAT). Panel B shows the results for physical activity (IPAQ) and physical capacity (6-MWD). Panel C shows the results for lung function in terms of forced vital capacity (FVC) and intrathoracic gas volume (ITGV). It can be seen that most unadjusted differences were either reduced or even inverted after the adjustments that took into account differences in patients characteristics. For PHQ-9 even a significant reduction was observed after matching, suggesting a beneficial effect of triple therapy

The third and fourth columns of Table 4 show the results of the optimal full matching procedure and the analysis using IPW, respectively. Part of the results is illustrated in Fig. 1. The matching was successful for both approaches as indicated by the fact that all mean differences of predictors were < 0.1 after matching. Again, the magnitude of differences was reduced or reversed in sign compared to the unadjusted differences. Moreover, most of the differences for the two approaches were similar to each other and similar to those of the multiple regression approach. Interestingly, the difference in PHQ-9 not only reversed in sign but became statistically significant with full matching (*p* < 0.05), in agreement with tendencies (*p* < 0.10) seen for adjusted regression and IPW. The unadjusted difference regarding 6-MWD, as an overall indicator of physical capability, virtually vanished after adjustment, while the difference in ITGV %predicted, as indicator of lung hyperinflation, reversed its sign. Moreover, the tendency for the single questions of CAT to show deterioration in the TA group, reversed its sign for most or all items, depending on the statistical method used. There were also changes in sign regarding the SGRQ impact and symptoms score.

### Associations between triple therapy and health care costs

Table 5 provides the results of the different approaches regarding health care costs as outcomes, in a similar manner as Table 4. Consistent over all three approaches to adjustment, significantly elevated costs remained for medication and specifically respiratory medication. The same was true for the tendency regarding the costs of physiotherapy. Remarkably, the estimate of the costs of hospitalization was much reduced after adjustment and no more significant between treatment groups. The same applied to the overall direct costs.

**Table 5:** Pre-defined outcome measures in terms of health care costs analyzed by different statistical approaches regarding the comparison “Triple always” versus “Triple not always”

Annual health care costs (€)		Univariate	Linear Regression	Optimal full matching	IPW
**Total direct costs**	Estimate	2843.1	1419.4	1025	1526.9
	95%CI	608.99; 5077.15	-853.5; 3680.6	-1476.0; 3526.0	-752.6; 3806.4
	*p* value	**0.012**	0.222	0.423	0.19
**Medication costs**	Estimate	977.8	806.8	748.2	808
	95%CI	519.51; 1436.17;	328.9; 1282.2	234.9; 1261.5	305.9; 1310.2
	*p* value	**< 0.001**	**0.001**	**0.005**	**0.002**
– **Respiratory medication costs**	Estimate	592.3	532.1	525.3	535.0
	95%CI	466.53; 717.99	411.1; 652.5	395.0; 655.5	400.9; 669.1
	*p* value	**< 0.001**	9.21e-16	**< 0.001**	**< 0.001**
– **Other medication costs**	Estimate	386.4	275.5	223.5	273.7
	95%CI	36.11; 808.88	-174.6; 723.4	-247.9; 694.9	-187.5; 734.9
	*p* value	0.073	0.231	0.354	0.246
**Outpatient costs**	Estimate	36.8	2.6	17.5	-7.7
	95%CI	106.84; 180.49	-151.9; 156.3	-151.3; 186.3	-167.8; 152.3
	*p* value	0.614	0.973	0.839	0.925
**Inpatient costs**	Estimate	1126.3	418.4	82.6	318.1
	95%CI	40.98; 2211.65	-673.9; 1505.2	-1271.4; 1436.6	-876.1; 1512.3
	*p* value	**0.042**	0.454	0.905	0.602
**Rehabilitation costs**	Estimate	113.4	7.04	-39.4	23.9
	95%CI	87.04; 313.88	-199.9; 213.0	-304.6; 225.8	-195.4; 243.1
	*p* value	**0.266**	0.947	0.771	0.831
**Physiotherapy**	Estimate	48.1	49.6	36.84	48.1
	95%CI	6.904; 89.280	3.5; 95.4	-14.4; 88.0	-1.7; 94.6
	*p* value	**0.022**	**0.036**	0.16	**0.043**

*IPW* Inverse Probability Weighting. All variables shown were first tested in univariate analyses and then in the multivariable analyses shown in the other three columns, always using the set of predictors shown in Table 1

## Discussion

The present study comprised an analysis of observational data from patients included in the COSYCONET observational cohort with COPD of GOLD categories C/D, in whom triple therapy is considered adequate. Triple therapy had to be present at two study visits separated by 18 months, which was defined as “always” versus the complementary “not always”. Compared to the complementary group, patients with triple therapy showed several impairments in clinical and functional characteristics. After adjustment by three different statistical approaches, the differences in the COPD characteristics chosen as outcome measures were no more significant or reversed in sign. The results regarding the depression score PHQ-9 even suggested an improvement by triple therapy. This seems relevant, as this score is highly sensitive to the severity of COPD as shown previously [28]. Regarding health care costs, unadjusted costs were higher in patients with triple therapy, but after adjustment for clinical and functional parameters, differences in costs were lower. Taken together, these findings indicate that the impairments naïvely found in patients with triple therapy under observational circumstances, largely disappeared or were reduced after adjustment for differences in clinical state. This seems remarkable as the clinical state was assessed in the presence of continuous triple therapy, thereby probably limiting the amount of possible adjustment. Our observations are compatible with data from clinical trials on benefits of triple therapy in GOLD groups C and D. They illustrate the potential of retrospective analyses of observational data but also underline their limits.

Triple therapy involving LABA, LAMA and ICS by separate or combined inhalers is in widespread use since a number of years. Previous data showed the strongest evidence in favor of ICS in patients with high frequency or severity of exacerbations. In the SPIROMICS cohort, up to 39% of GOLD grade 1/2 patients with CAT score levels < 10 had ICS plus bronchodilator therapy, whereas up to 67% of patients with higher symptom burden (CAT > 10) had this combination. ICS treatment, however, has also been found in many patients not fulfilling the GOLD C/D criteria [13], in line with investigations showing that 32% of all COPD patients in primary care end up with triple therapy within 12 months from initial diagnosis [29]. In the present analysis, we used the fact that at the time of data collection recommendations on the use of triple therapy [30] were very similar to the recent ones [1]. To identify factors associated with triple therapy, we used a broad panel of questionnaires and functional measures, in the expectation to cover some of the underlying determinants with this panel, which was much broader than the assessments commonly used in daily practice.

When COSYCONET started enrollment in 2010, none of the currently available single-inhaler triple therapies was available. To date, one study has shown that single-inhaler triple therapy (beclomethasone-dipropionate/formoterol/glycopyrronium) was equivalent to triple therapy in separate inhalers, even with different LAMA components [31]. No head-to-head comparisons of other single-inhaler triple therapies versus the use of free-combination triple therapy are available so far. We cannot exclude that this had an impact on the results of our analysis, since neither inhalers nor formulations were standardized. At least, a previous analysis indicated that the overall adherence to inhalation therapy in COSYCONET patients was very high [22].

Triple therapy has been linked to survival benefits in ETHOS and IMPACT study [5, 6], thus we also looked for such effects in the present data. However, sample size and event numbers were too small to allow for a meaningful analysis, despite an observation period of up to 72 months. It might also be of interest that in the ETHOS and IMPACT studies [5, 6] mortality was a secondary endpoint, for which a positive effect was observed for the first 3 months, although the effect might somehow depend on the length of follow up [32]. Our data indicate that in COSYCONET patients with greater severity of COPD and probably greater mortality risk were more often treated with triple therapy. As we aimed to investigate potential differences in clinical and functional state in relation to continuous triple therapy, we averaged over two study visits covering a period of 18 months. Moreover, all patients were already in the high exacerbation category C or D (recently summarized as E) at both visits. Thus, by the design of the present analysis, no conclusions on potential acute effects on exacerbations and mortality are possible, different from randomized controlled trials.

We employed three different approaches of adjustment for clinical and functional characteristics. These approaches yielded similar results, with only small variations that could be attributed to specific characteristics of the adjustment procedures. The main results were that most of the significant differences that appeared in a naïve comparison of treatment groups, were largely reduced and non-significant after adjustment. For adjustment, we primarily used functional parameters that indeed differed between the two treatment groups. As outcome variables, we primarily used scores describing the patients’ clinical state, as well as a few functional parameters, for example blood gases, that are unlikely to have played a role in the prescription of triple therapy. Comorbidities were balanced between both groups and not further analyzed. The differences in ITGV and FVC, as indicators of lung volume, were no more significant after adjustment and suggested a tendency towards improvement with triple therapy. The same was true for the 6-MWD. Regarding the depression score PHQ 9, even an improvement with triple therapy was observed after matching. Although this was a purely observational result based on post hoc-matching and thus should be considered with some caution, it might indicate a true improvement, particularly when regarding the fact that this score is highly sensitive to indices describing COPD characteristics [28]. We analyzed the CAT score in terms of its single items, as from previous results we expected them to carry different information [28, 33, 34]. There were some hints on small effects of triple therapy on the CAT items, while the SGRQ activity score that was impaired in triple therapy patients without adjustment, was much reduced after matching. This is of interest, as this score integrates over a range of limitations occurring and being relevant in COPD. This result was consistent with the small reductions in CAT items 5 and 6, which also address activity.

The COSYCONET study collected detailed data on medical costs, however without information on the reasons for hospitalization and associated costs. Without adjustment, total direct costs, medication costs, specifically for respiratory medication, as well as inpatient and rehabilitation costs were higher in the triple group. After adjustment, total direct costs were still elevated but no more different between treatment groups. This could be mainly attributed to the reduction in inpatient costs, as total medication and respiratory medication costs were only slightly reduced. The contributions from rehabilitation and physiotherapy were always small. These observations again suggest beneficial effects of triple therapy, which, interestingly enough, were not based on medication costs but on costs for inpatient treatment, again consistent with the assumption of an improvement in clinical state. The relatively large reduction in inpatient costs might also be taken as a hint that a possible risk from pneumonia that has been repeatedly described for ICS therapy [3, 4, 35, 36] did not play a major in our data.

### Limitations

The cross-sectional, observational nature of our data only allowed for post hoc statistical adjustment as an attempt to make the two treatment groups comparable. This was not an interventional study allowing for practical consequences and recommendations, as it aimed at understanding of the factors related to triple therapy under real-world conditions. In the meantime, it became known that beneficial effects of ICS on COPD exacerbations are linked to an elevated number of circulating eosinophils, while in patients with low blood eosinophils counts eosinopenia raises the risk of pneumonia [37–39]. At the time of the study visits, differential blood counts were not part of regular assessments in COSYCONET, thus an analysis taking into account blood eosinophils was not feasible. At the time of recruitment, inhaled steroids were generally used earlier in the treatment of COPD patient than according to recent recommendations. This and the observational character of the study explain why in this study some COPD patients were treated with ICS monotherapy although this does not reflect the current treatment recommendations. Another limitation of the study is that the reasons for hospital admission were not recorded, so the results can only be interpreted in part.

## Conclusion

Randomized controlled intervention trials suggest benefits from triple therapy in patients with COPD and high risk of exacerbations. We studied whether such effects could be detected under continuous medication in patients with stable COPD under observational circumstances closer to real-life conditions. This required statistical adjustment methods such as matching or weighting. Without adjustment, patients with triple therapy showed significant impairments in parameters of clinical and functional state, as well as treatment costs. After adjustment, most of the differences were reduced or even reverted and no more statistically significant, except for medication costs. These findings are consistent with the assumption of beneficial effects of triple therapy under continuous, long-term treatment. However, in relation to other comparisons between controlled and observational results that have been performed, they demonstrate the increasing difficulty to verify medication effects in a non-randomized, observational setting in severely ill COPD patients using different devices and formulations of triple therapy.

### Supplementary Information

**Supplementary Materials 1. **

## Data Availability

Data may be obtained from a third party and are not publicly available. The full dataset supporting the conclusions of this article is available upon request and application from the Competence Network Asthma and COPD (ASCONET, http://www.asconet.net/html/cosyconet/projects), alternatively the datasets used and/or analysed during the current study are available from the corresponding author on reasonable request.
