# Supplementary material for: Effects of triple therapy on disease burden in patients of GOLD groups C and D: results from the observational COPD cohort COSYCONET
Source: BMC Pulm Med. 2024 Mar 1;24:103. doi: 10.1186/s12890-024-02902-4 (PMC10905841; doi:10.1186/s12890-024-02902-4)
Supplement: Supplementary file 1 — Supplementary Materials 1. [file 12890_2024_2902_MOESM1_ESM.docx]

| **Therapy options** | **GOLD C** | **GOLD D** | **All** |
| --- | --- | --- | --- |
| Number | 89 | 169 | 258 |
| **Always LAMA** |  |  |  |
| yes | 68 (76.4%) | 148 (87.6%) | 216 (83.7%) |
| no | 21 (23.6%) | 21 (12.4%) | 42 (16.3%) |
| **Always LABA** |  |  |  |
| yes | 81 (91.0%) | 158 (93.5%) | 239 (92.6%) |
| no | 8 (9.0%) | 11 (6.5%) | 19 (7.4%) |
| **Always LAMA+LABA** |  |  |  |
| yes | 65 (73.0%) | 142 (84.0%) | 207 (80.2%) |
| no | 24 (27.0%) | 27 (16.0%) | 51 (19.8%) |
| **Always ICS** |  |  |  |
| yes | 65 (73.0%) | 128 (75.7%) | 193 (74.8%) |
| no | 24 (27.0%) | 41 (24.3%) | 65 (25.2%) |
| **Always LAMA+ICS** |  |  |  |
| yes | 52 (58.4%) | 115 (68.0%) | 167 (64.7%) |
| no | 37 (41.6%) | 54 (32.0%) | 91 (35.3%) |
| **Always LABA+ICS** |  |  |  |
| yes | 63 (70.8%) | 125 (74.0%) | 188 (72.9%) |
| no | 26 (29.2%) | 44 (26.0%) | 70 (27.1%) |
| **Always Triple** |  |  |  |
| yes | 50 (56.2%) | 112 (66.3%) | 162 (62.8%) |
| no | 39 (43.8%) | 57 (33.7%) | 96 (37.2%) |

**Supplemental Table S1.** Distribution over various kinds of inhalation therapy shown for GOLD group C and D where “always” indicates that the therapy was present at both visits (1 & 3).
